# Supplementary figures and images for: Multiple ciliary localization signals control INPP5E ciliary targeting
Source: eLife. 2022 Sep 5;11:e78383. doi: 10.7554/eLife.78383 (PMC9444247; doi:10.7554/eLife.78383)

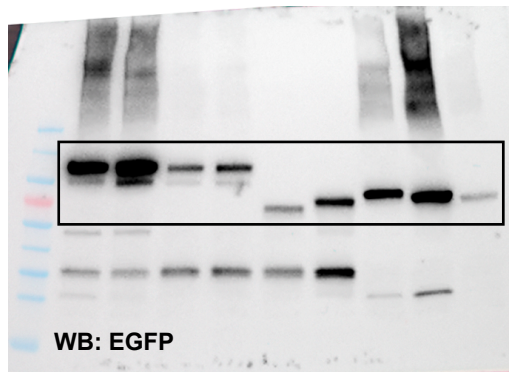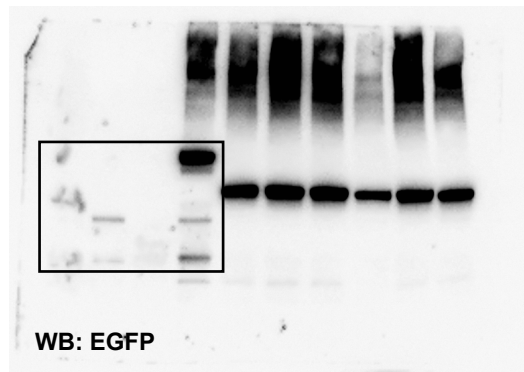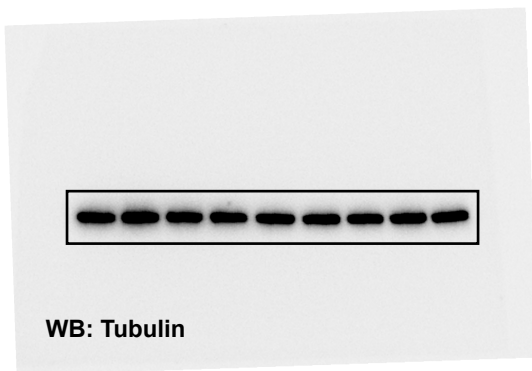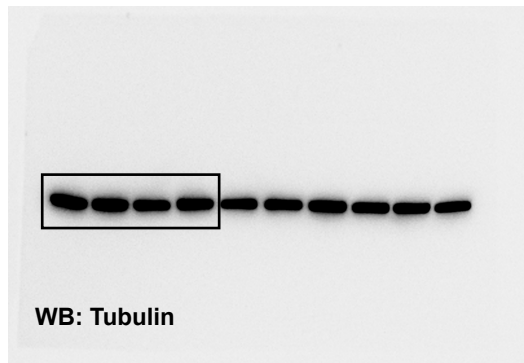

Supplement: Figure 1—figure supplement 1—source data 1. — Relevant bands are inside rectangles. See Figure 1—figure supplement 1 for more details. [file elife-78383-fig1-figsupp1-data1.pdf]

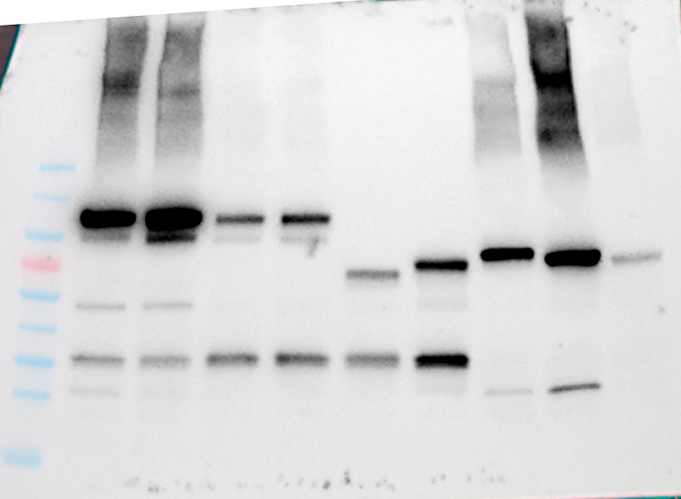

Supplement: Figure 1—figure supplement 1—source data 2. [file elife-78383-fig1-figsupp1-data2.tif]

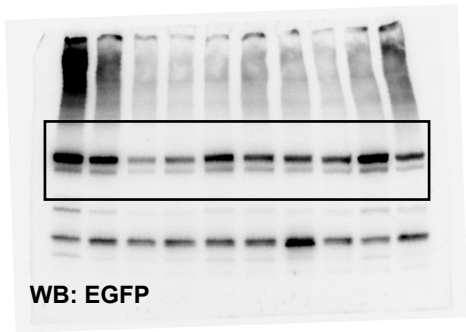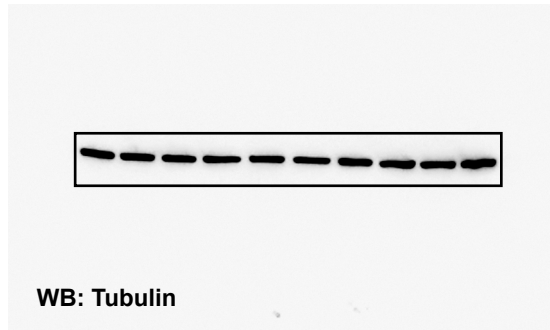

Supplement: Figure 2—figure supplement 2—source data 1. — Relevant bands are inside rectangles. See Figure 2—figure supplement 2 for more details. [file elife-78383-fig2-figsupp2-data1.pdf]

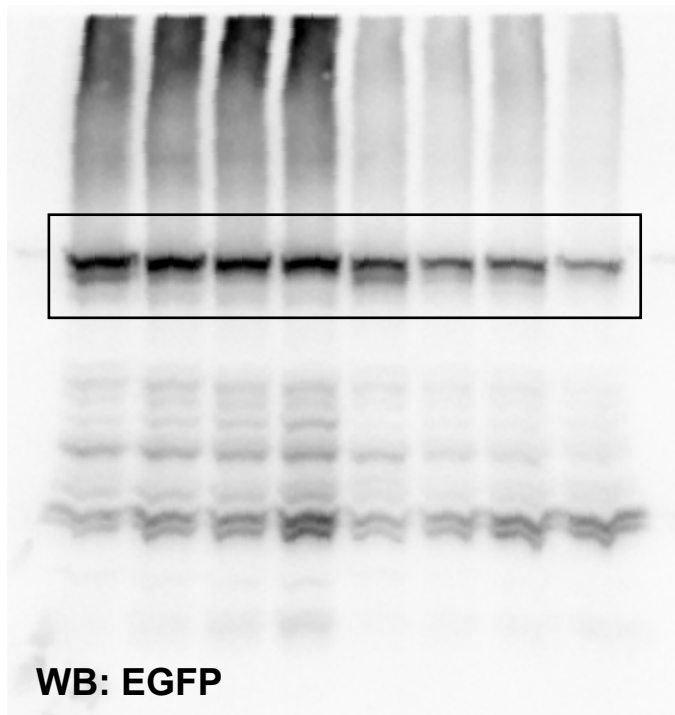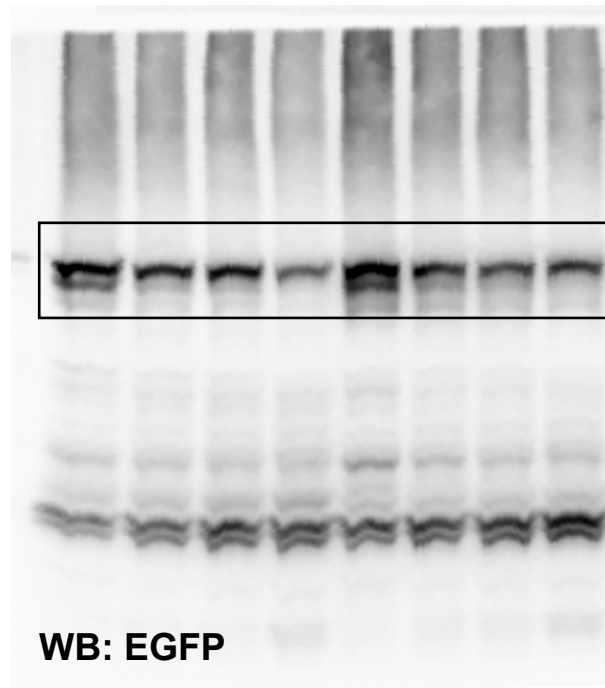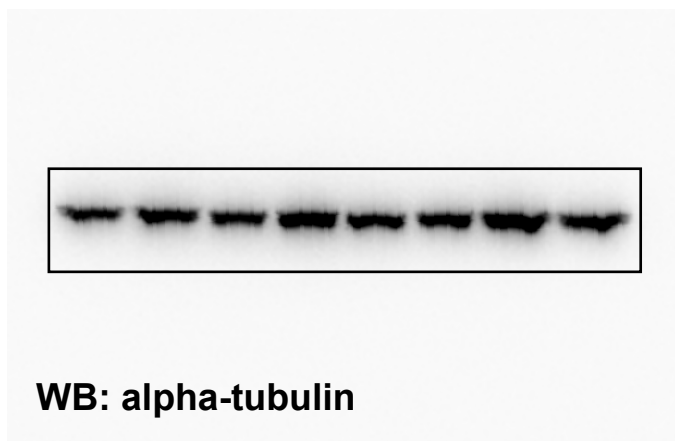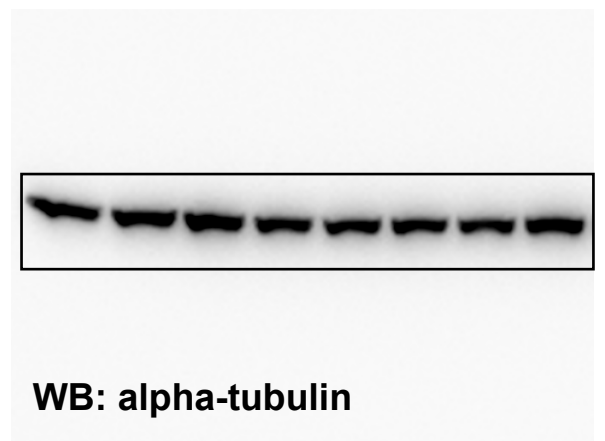

Supplement: Figure 2—figure supplement 3—source data 1. — Relevant bands are inside rectangles. See Figure 2—figure supplement 3 for more details. [file elife-78383-fig2-figsupp3-data1.pdf]

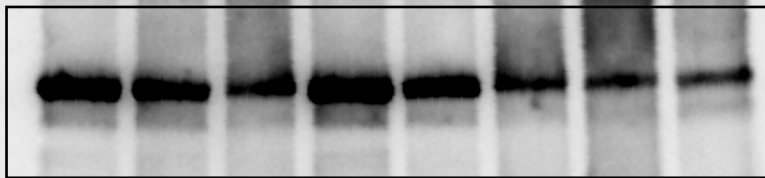

**WB: EGFP**

Supplement: Figure 2—figure supplement 4—source data 1. — Relevant bands are inside rectangle. See Figure 2—figure supplement 4 for more details. [file elife-78383-fig2-figsupp4-data1.pdf]

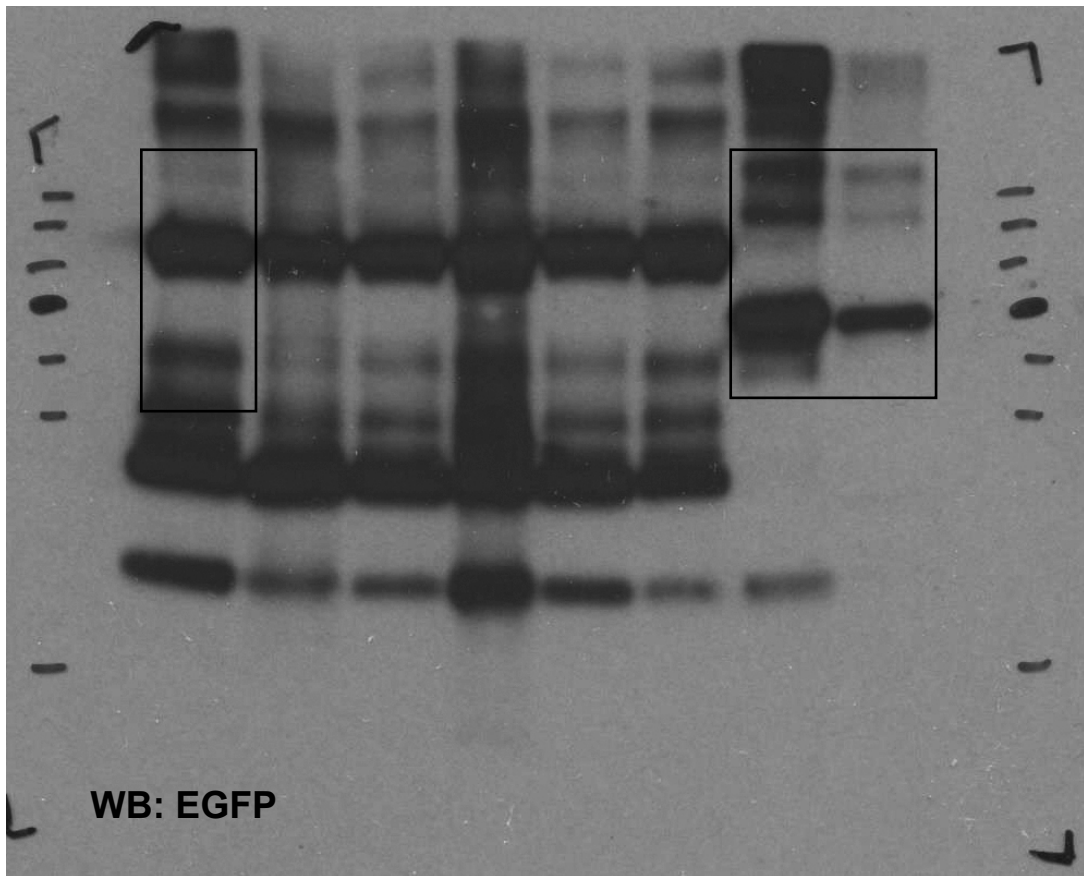

Supplement: Figure 3—figure supplement 1—source data 1. — Relevant bands are inside rectangles. See Figure 3—figure supplement 1 for more details. [file elife-78383-fig3-figsupp1-data1.pdf]

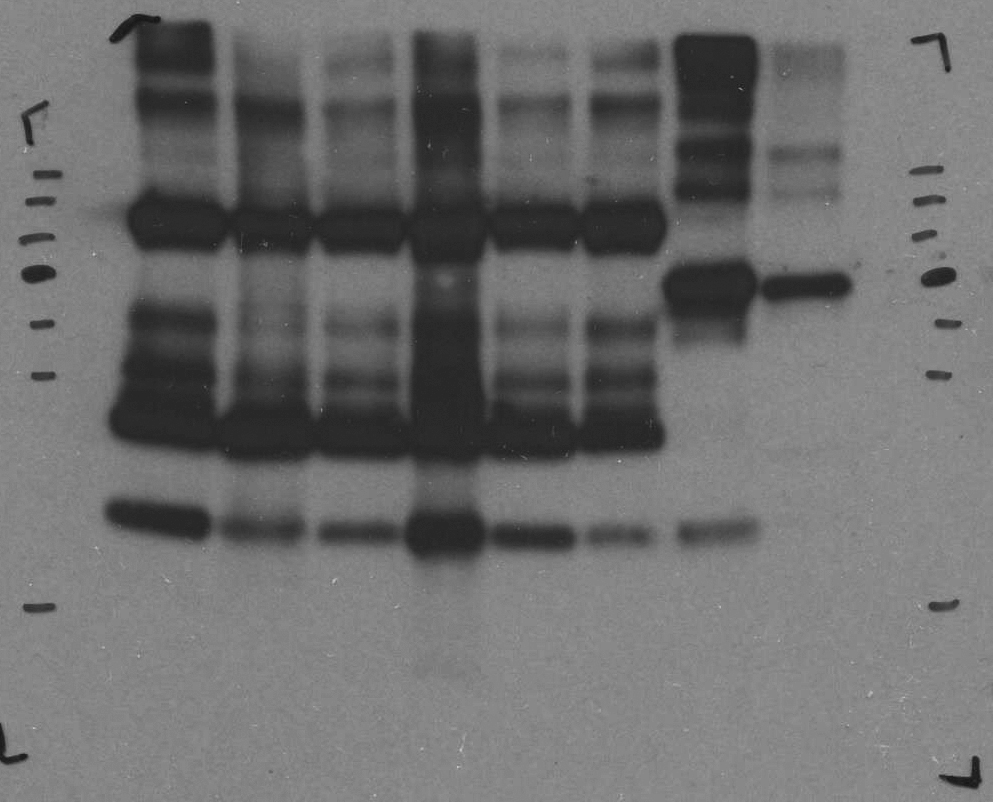

Supplement: Figure 3—figure supplement 1—source data 2. [file elife-78383-fig3-figsupp1-data2.tif]

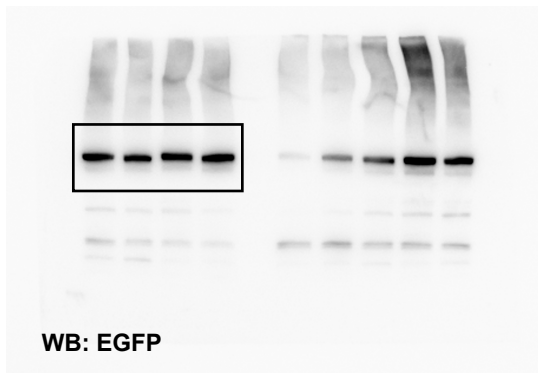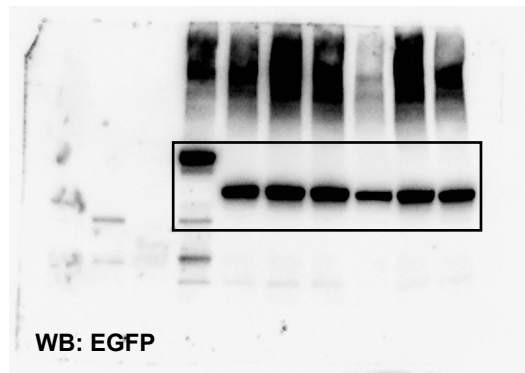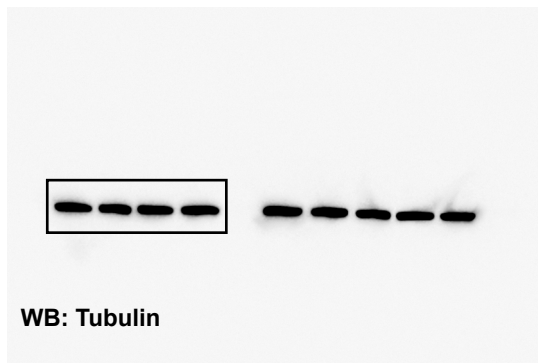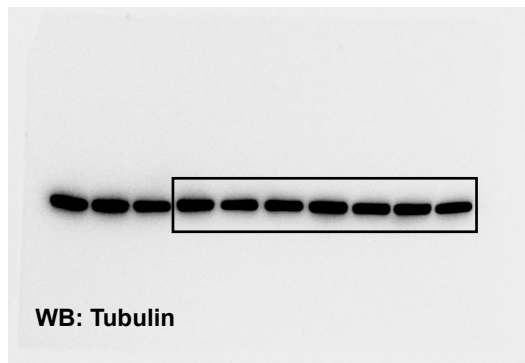

Supplement: Figure 3—figure supplement 2—source data 1. — Relevant bands are inside rectangles. See Figure 3—figure supplement 2 for more details. [file elife-78383-fig3-figsupp2-data1.pdf]

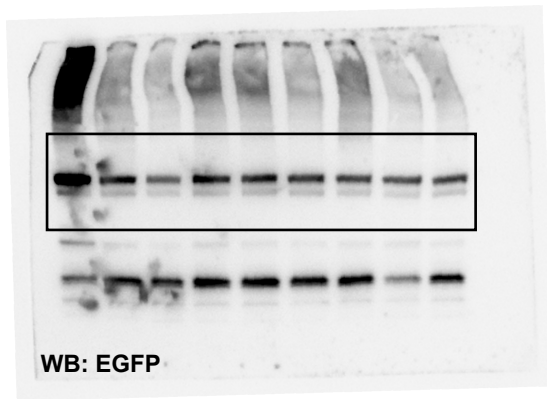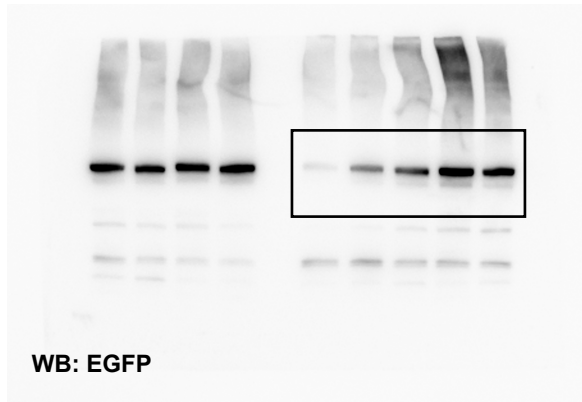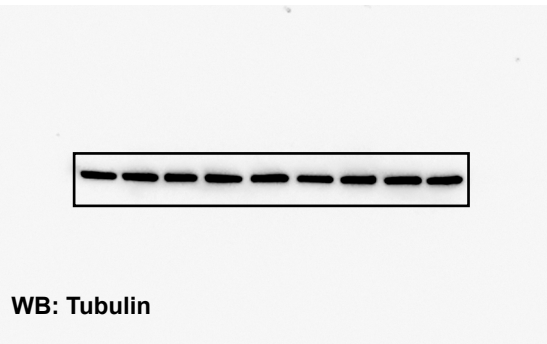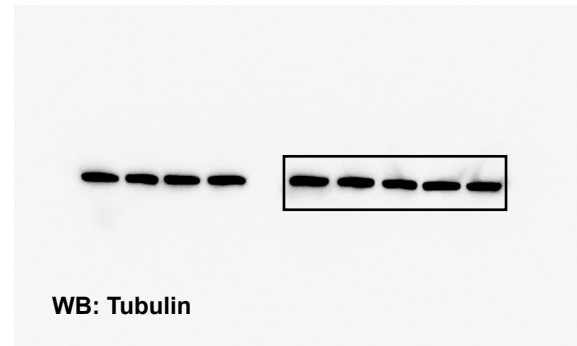

Supplement: Figure 5—source data 1. — Relevant bands are inside rectangles. See Figure 5 for more details. [file elife-78383-fig5-data1.pdf]

**a**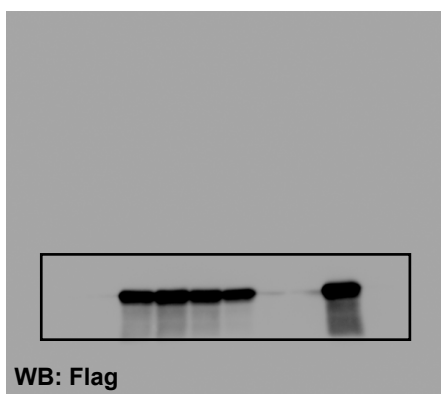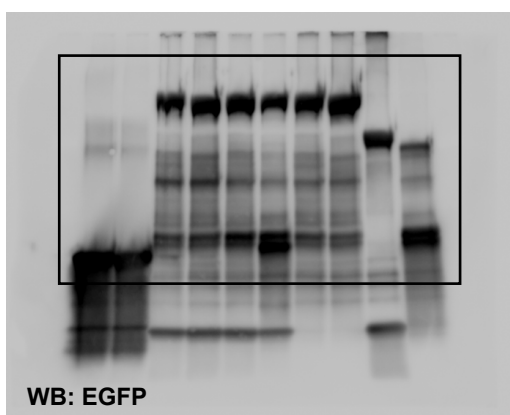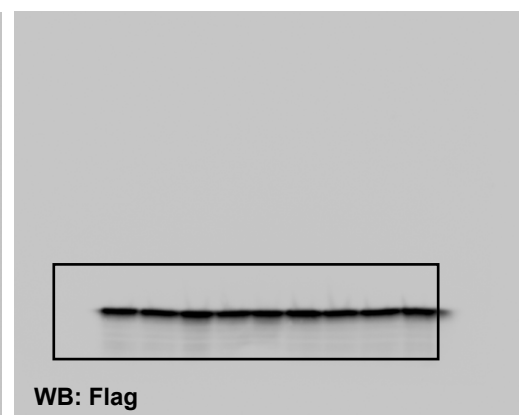**b**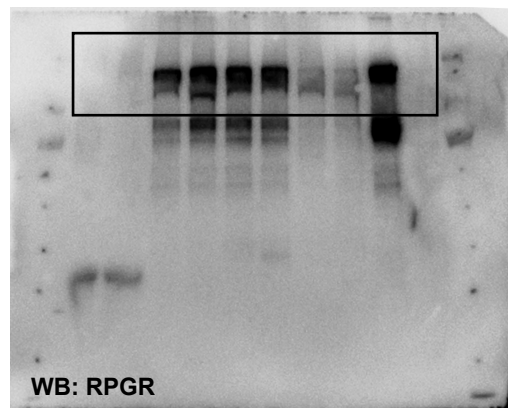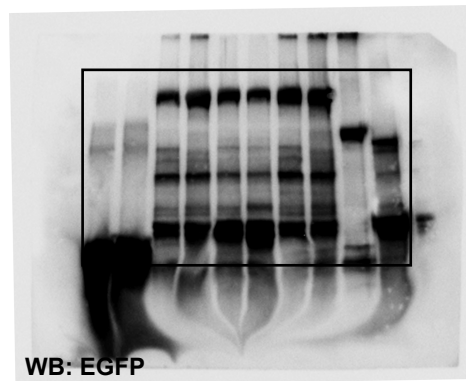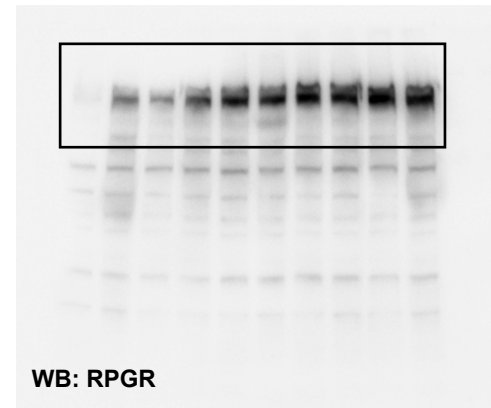**c**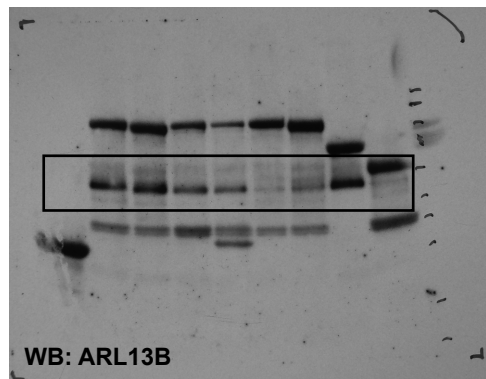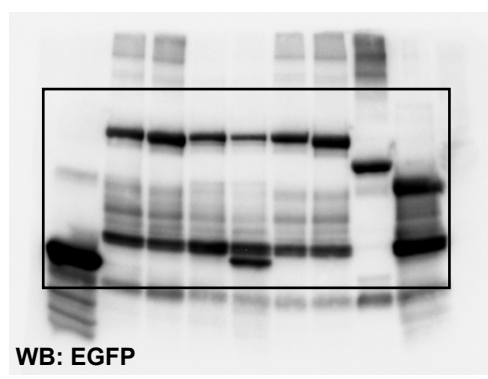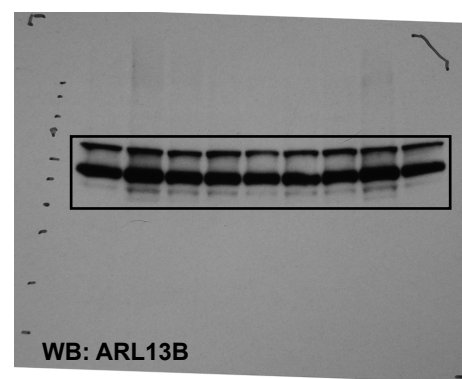

Supplement: Figure 6—source data 1. — Relevant bands are inside rectangles. (a) Immunoblots from Figure 6a. (b) Immunoblots from Figure 6b. (c) Immunoblots from Figure 6c (EGFP bands are also seen in ARL13B immunoblot on the left). See Figure 6 for more details. [file elife-78383-fig6-data1.pdf]

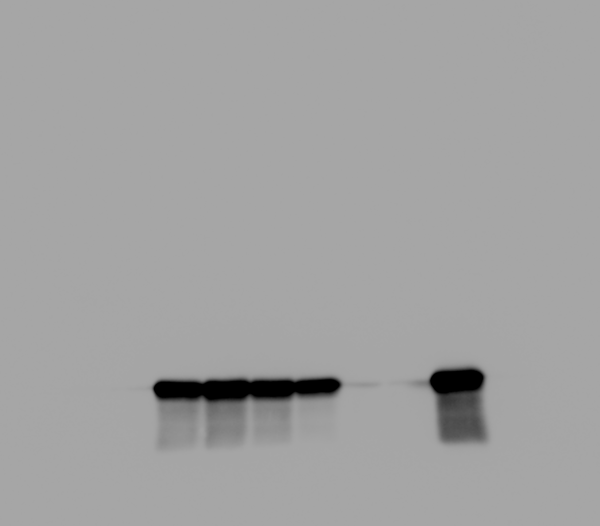

Supplement: Figure 6—source data 2. [file elife-78383-fig6-data2.tif]

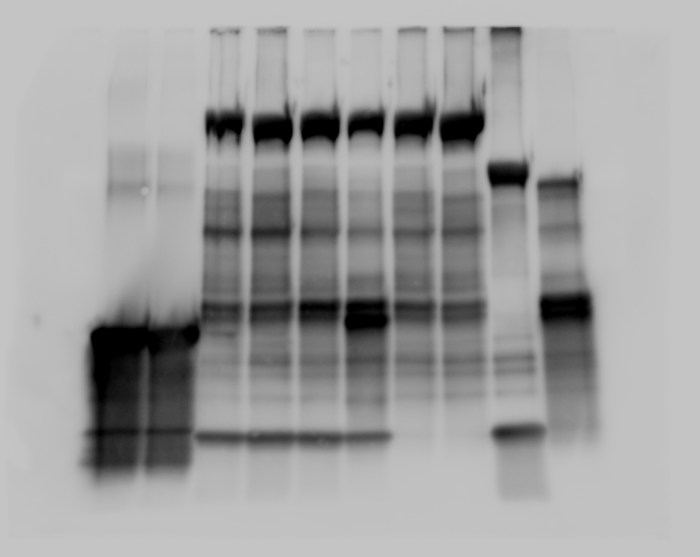

Supplement: Figure 6—source data 3. [file elife-78383-fig6-data3.tif]

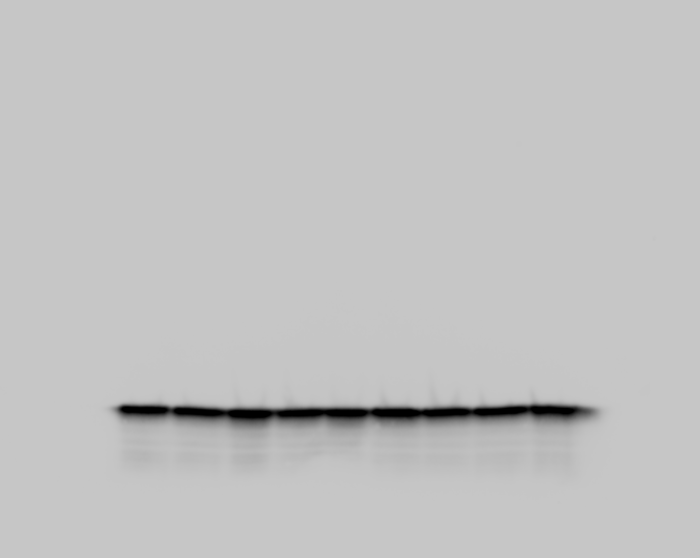

Supplement: Figure 6—source data 4. [file elife-78383-fig6-data4.tif]

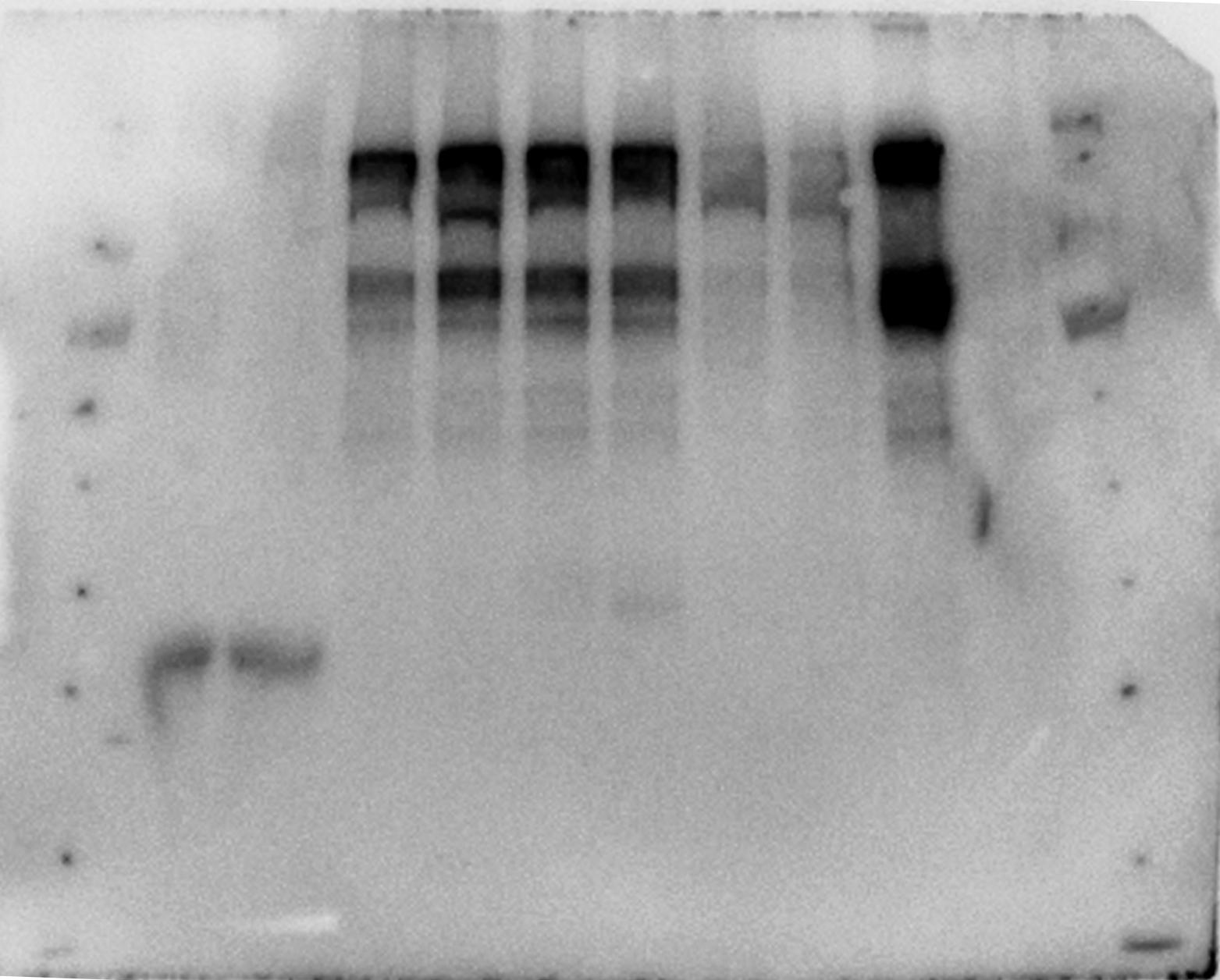

Supplement: Figure 6—source data 5. [file elife-78383-fig6-data5.zip › Figure 6-Source Data 5.Tif]

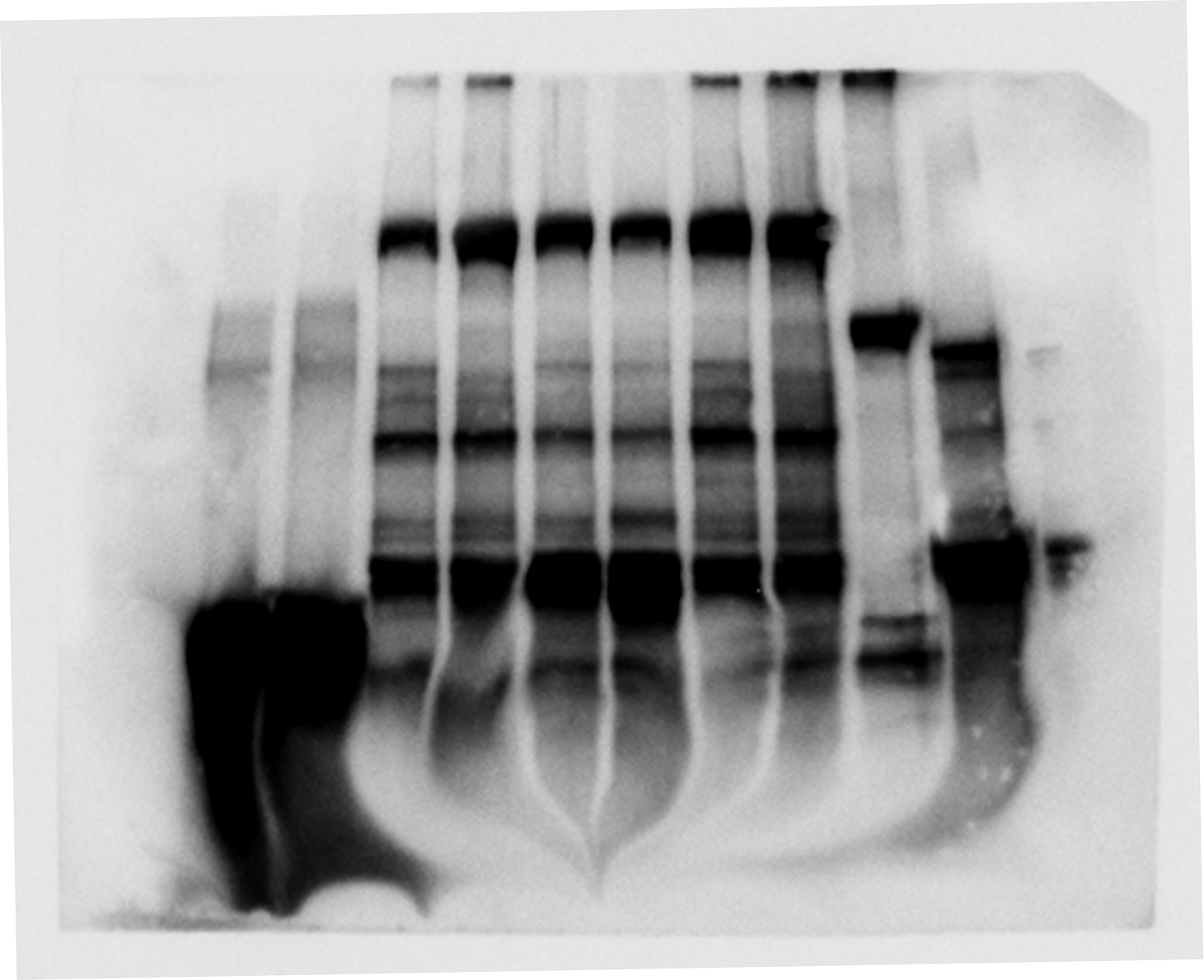

Supplement: Figure 6—source data 6. [file elife-78383-fig6-data6.zip › Figure 6-Source Data 6.Tif]

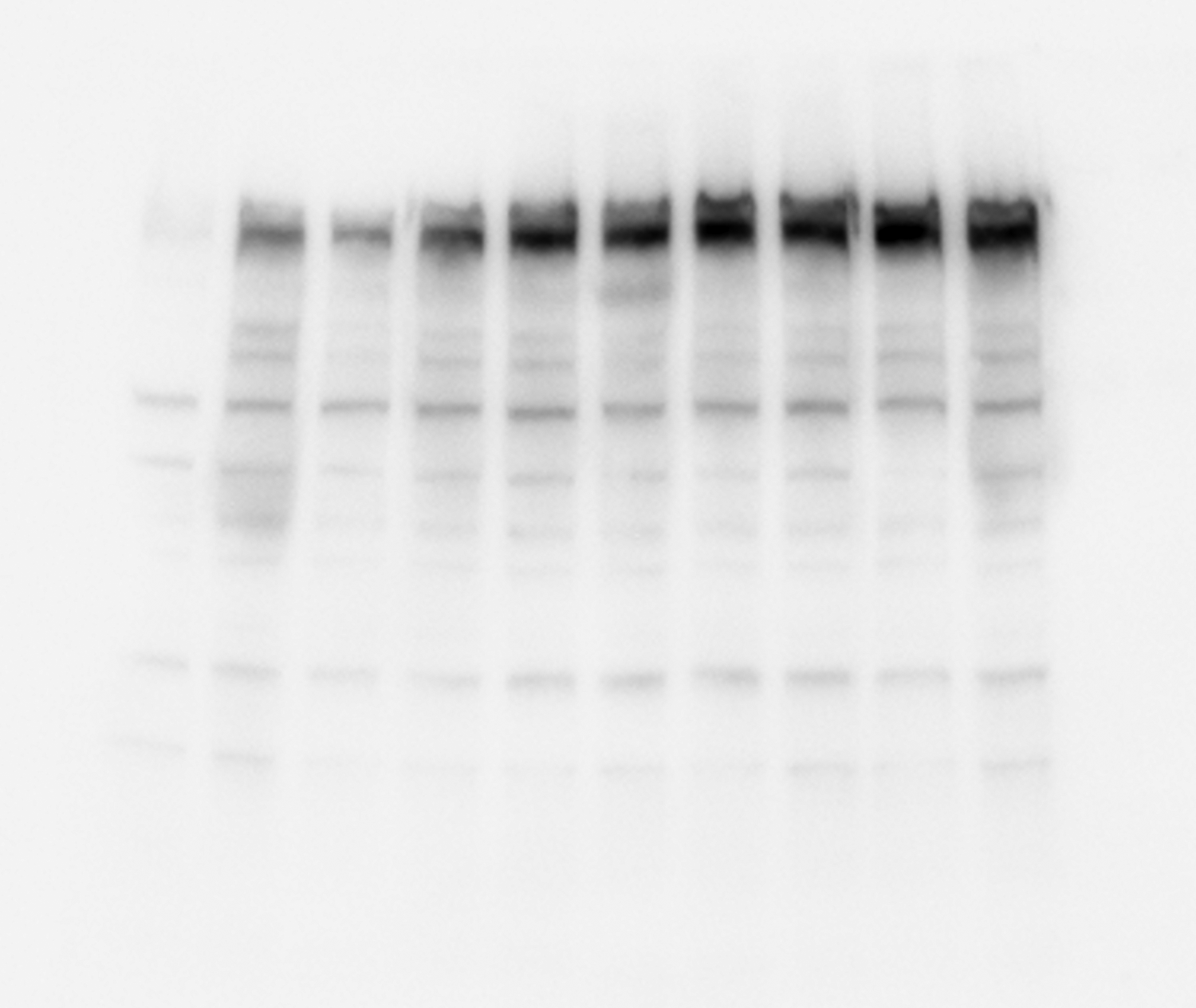

Supplement: Figure 6—source data 7. [file elife-78383-fig6-data7.zip › Figure 6-Source Data 7.Tif]

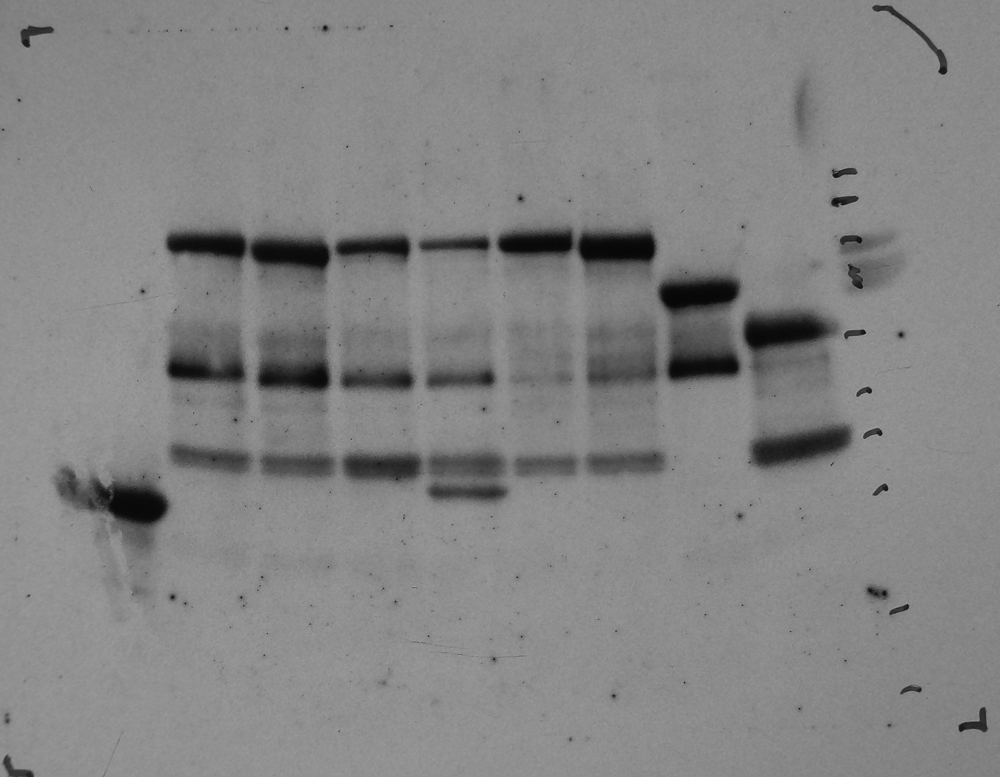

Supplement: Figure 6—source data 8. [file elife-78383-fig6-data8.tif]

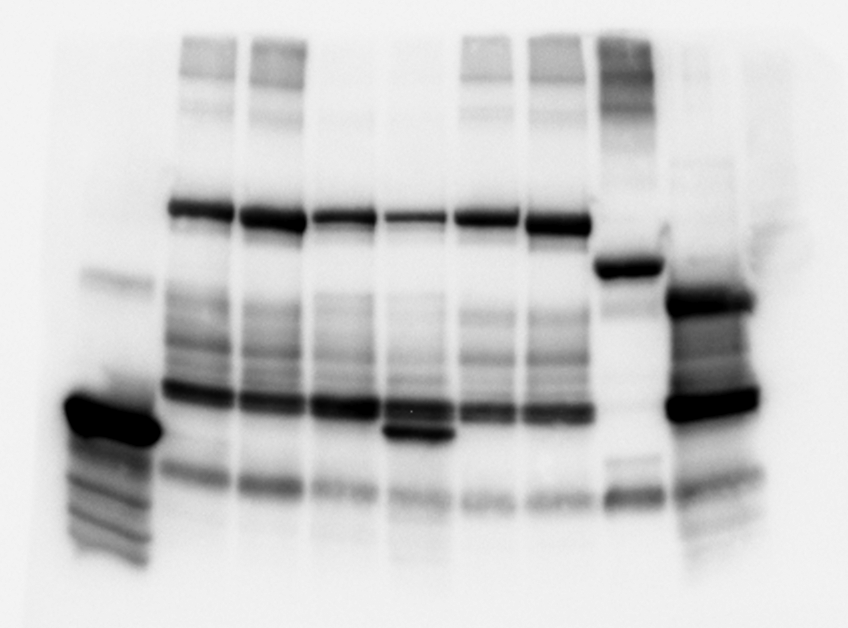

Supplement: Figure 6—source data 9. [file elife-78383-fig6-data9.zip › Figure 6-Source Data 9.Tif]

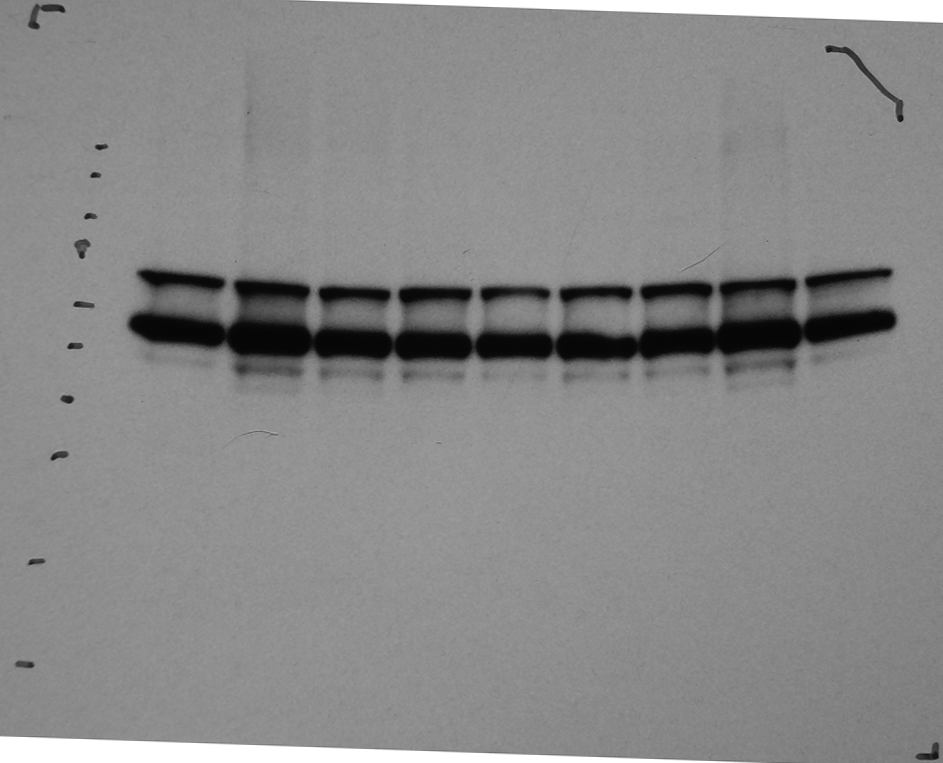

Supplement: Figure 6—source data 10. [file elife-78383-fig6-data10.zip › Figure 6-Source Data 10.Tif]

**a**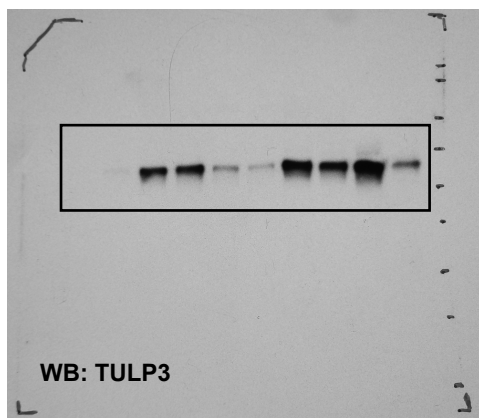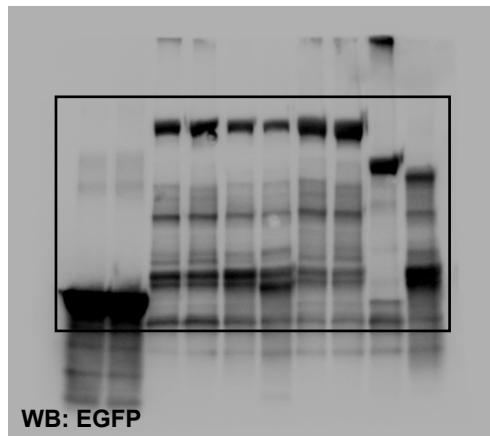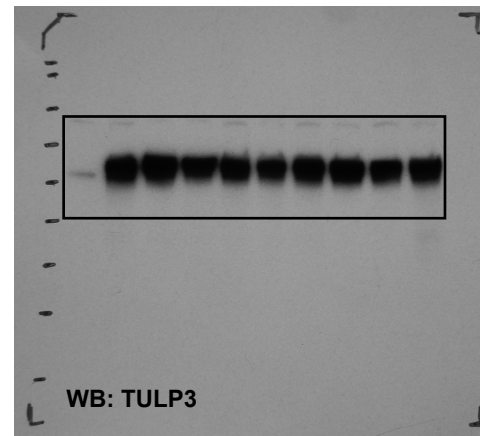**b**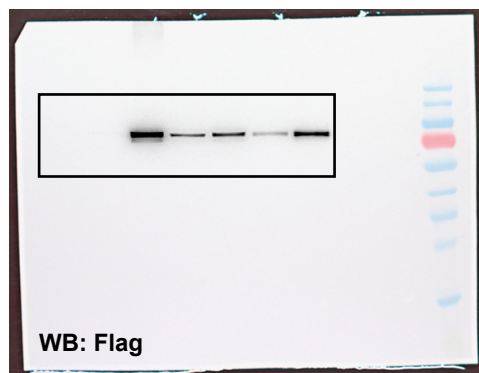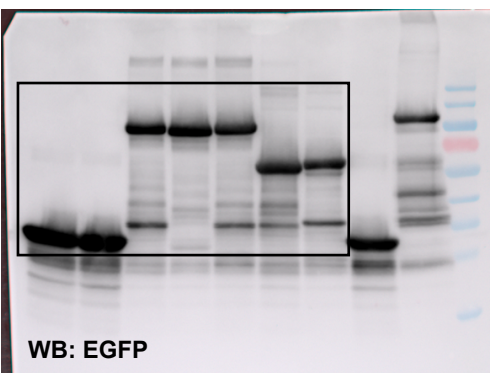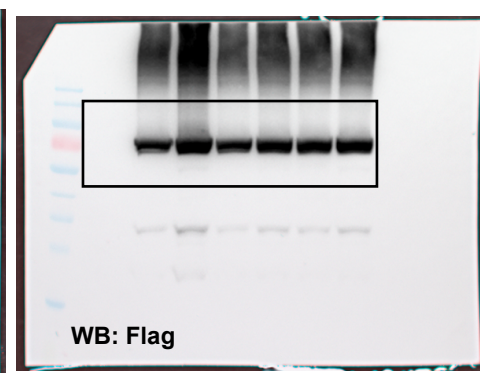**c**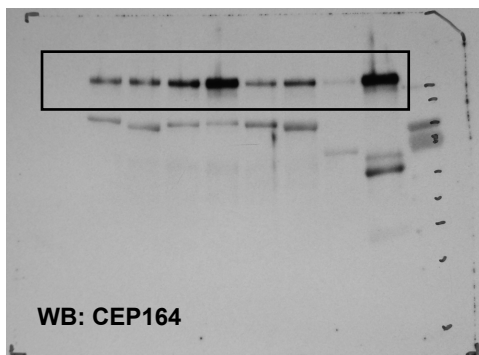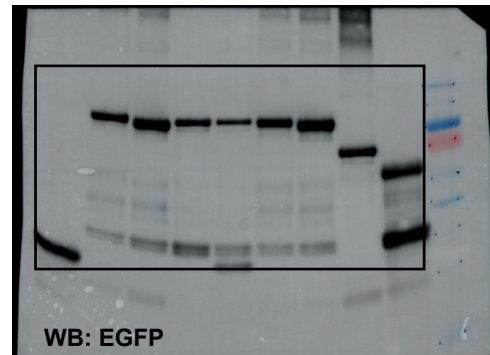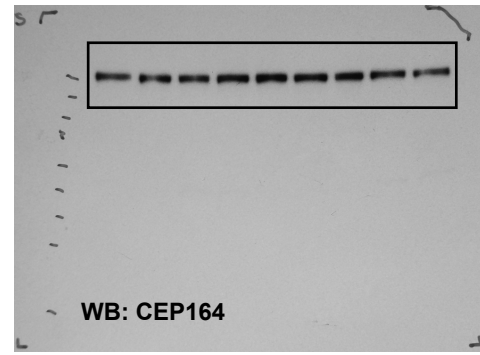**d**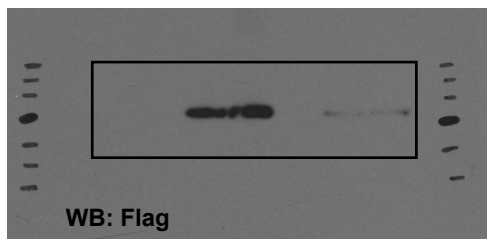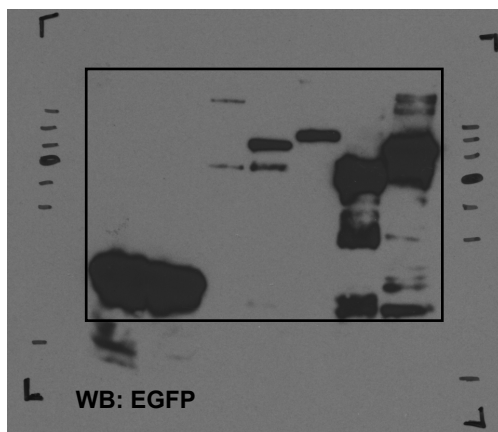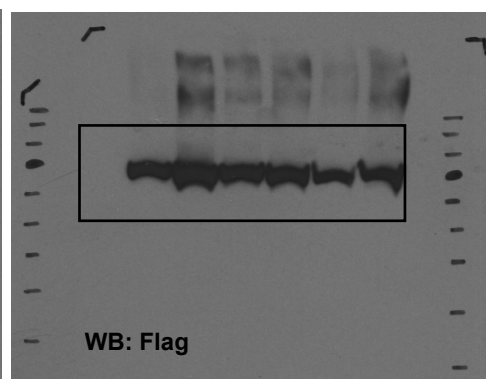

Supplement: Figure 7—source data 1. — Relevant bands are inside rectangles. (a) Immunoblots from Figure 7a. (b) Immunoblots from Figure 7c. (c) Immunoblots from Figure 7e (EGFP bands also visible in left CEP164 blot). (d) Immunoblots from Figure 7g. See Figure 7 for more details. [file elife-78383-fig7-data1.pdf]

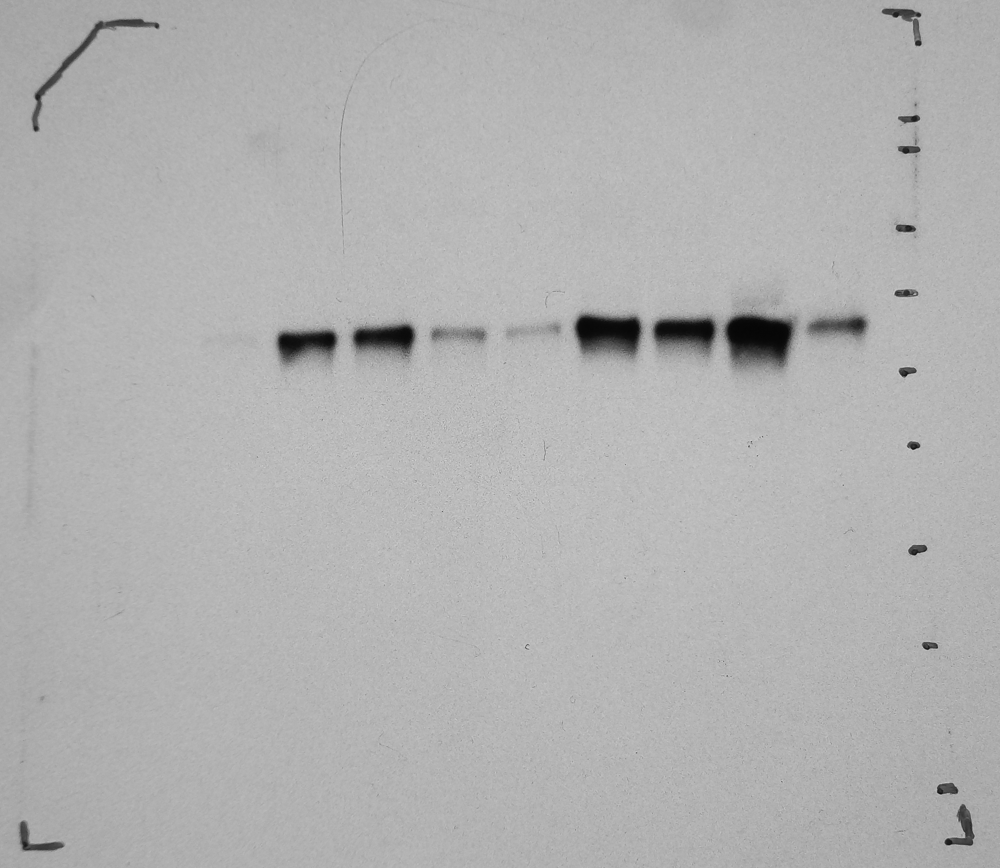

Supplement: Figure 7—source data 2. [file elife-78383-fig7-data2.tif]

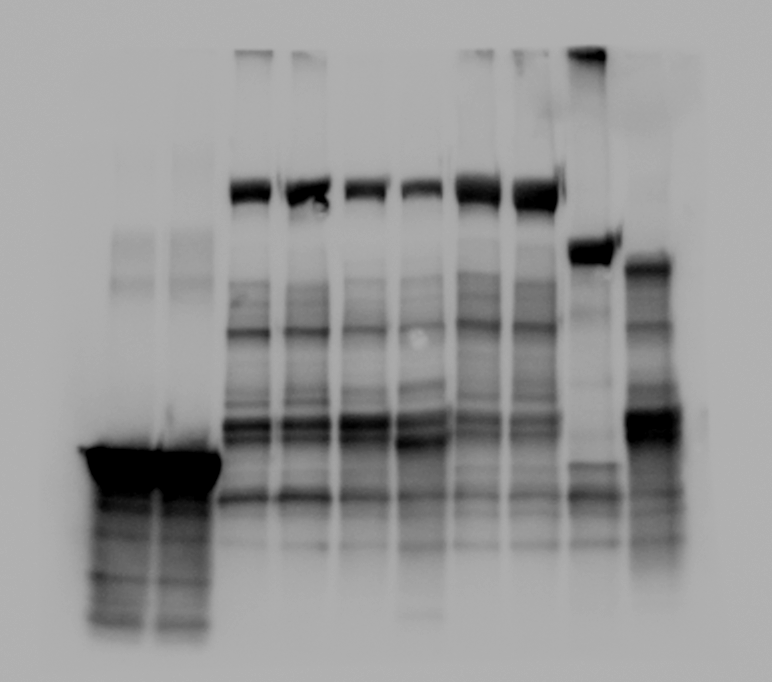

Supplement: Figure 7—source data 3. [file elife-78383-fig7-data3.zip › Figure 7-Source Data 3.Tif]

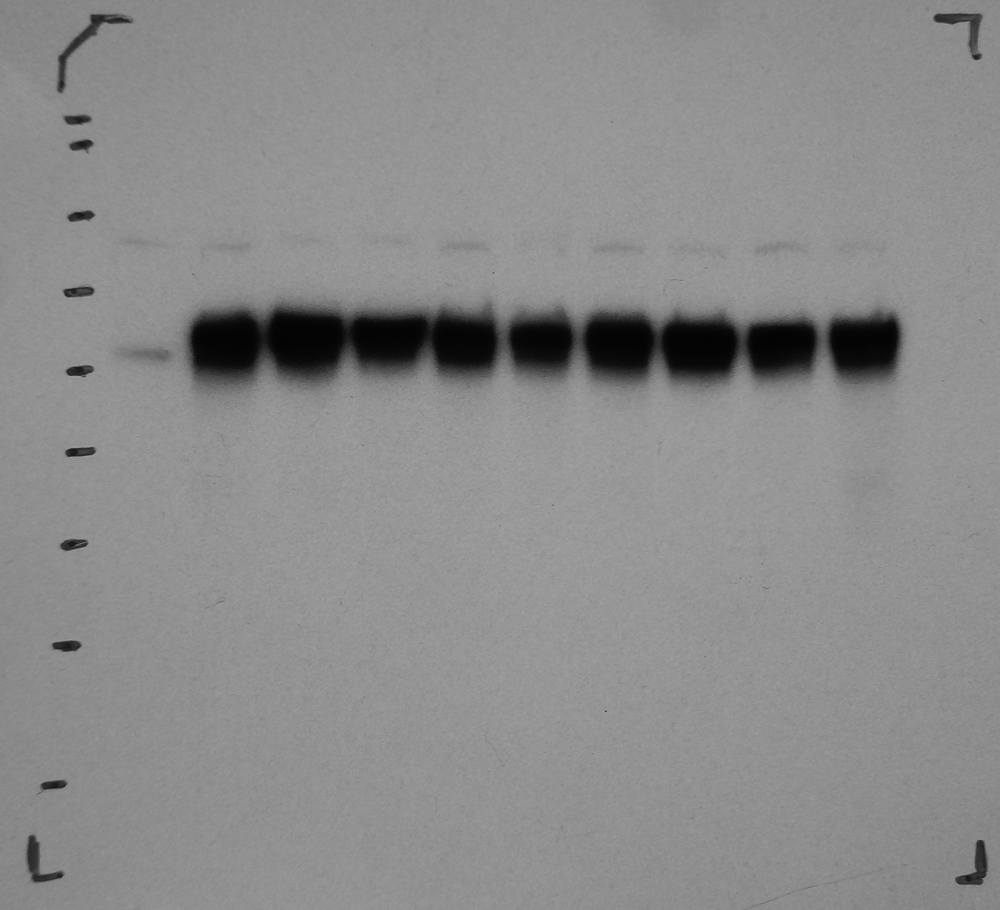

Supplement: Figure 7—source data 4. [file elife-78383-fig7-data4.tif]

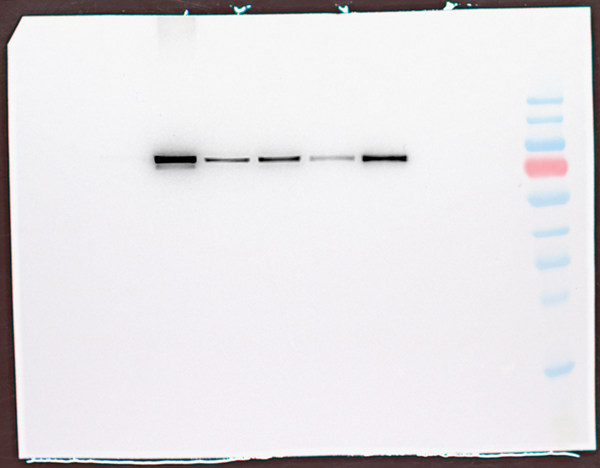

Supplement: Figure 7—source data 5. [file elife-78383-fig7-data5.zip › Figure 7-Source Data 5.Tif]

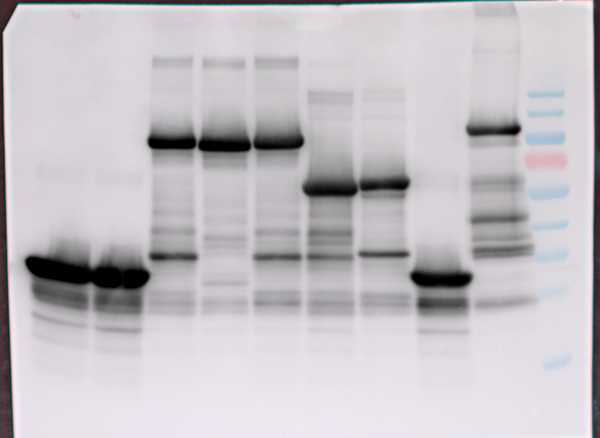

Supplement: Figure 7—source data 6. [file elife-78383-fig7-data6.zip › Figure 7-Source Data 6.Tif]

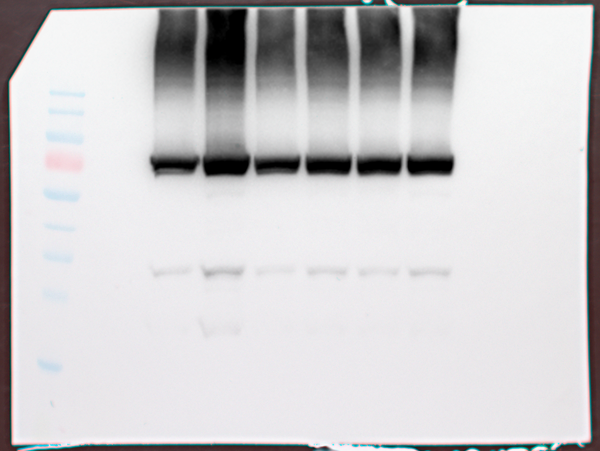

Supplement: Figure 7—source data 7. [file elife-78383-fig7-data7.tif]

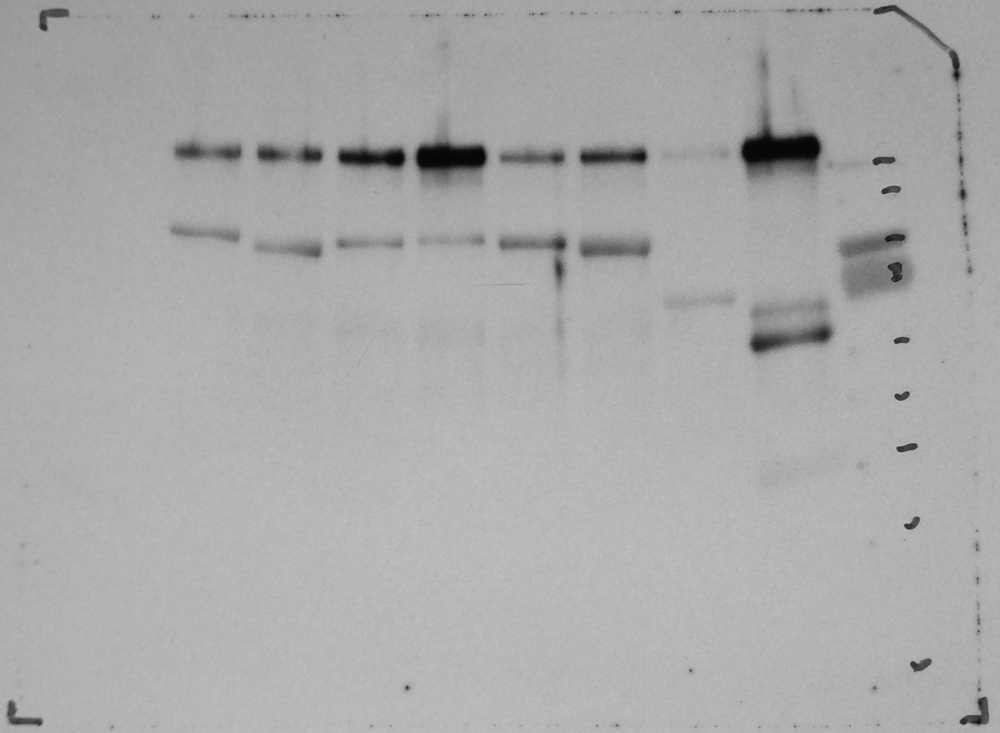

Supplement: Figure 7—source data 8. [file elife-78383-fig7-data8.tif]

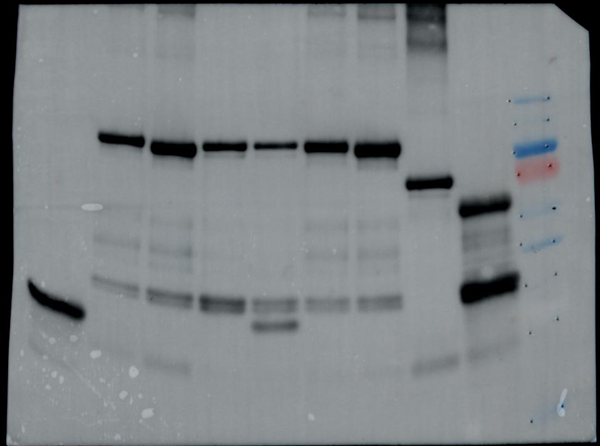

Supplement: Figure 7—source data 9. [file elife-78383-fig7-data9.tif]

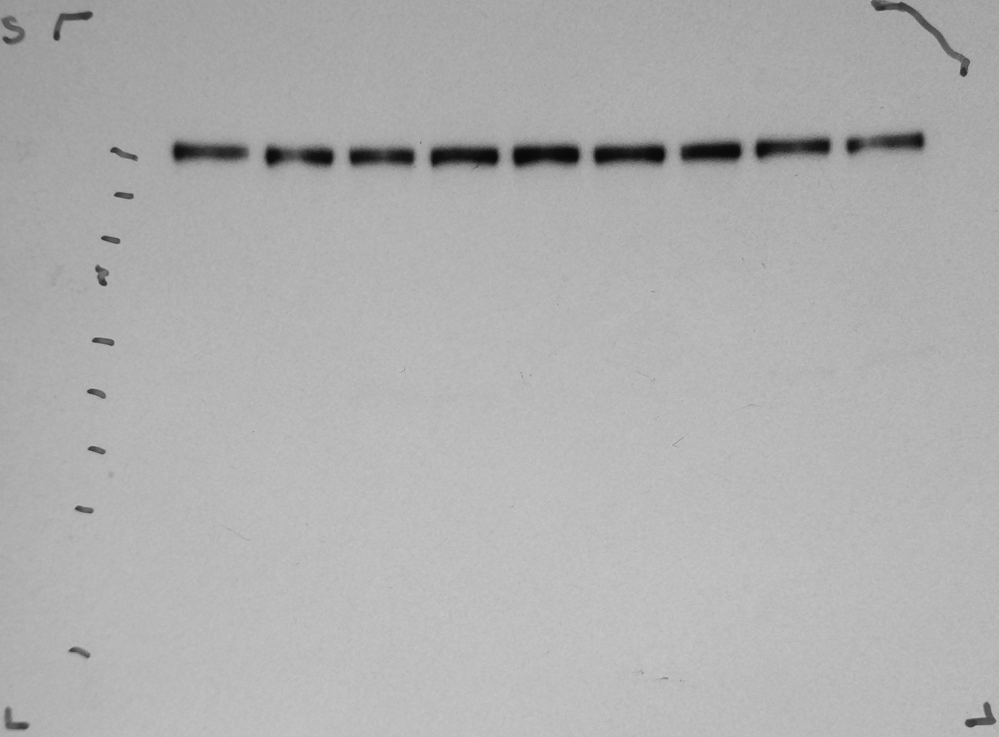

Supplement: Figure 7—source data 10. [file elife-78383-fig7-data10.tif]

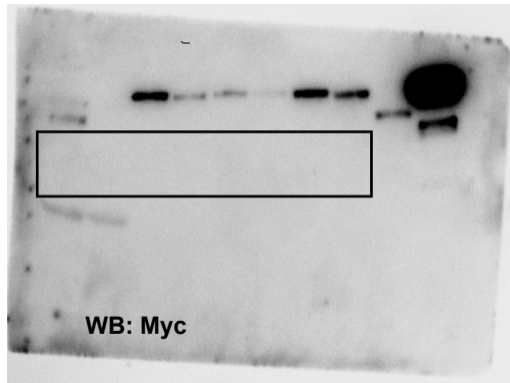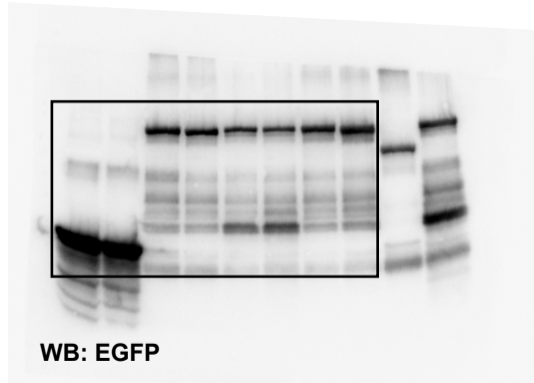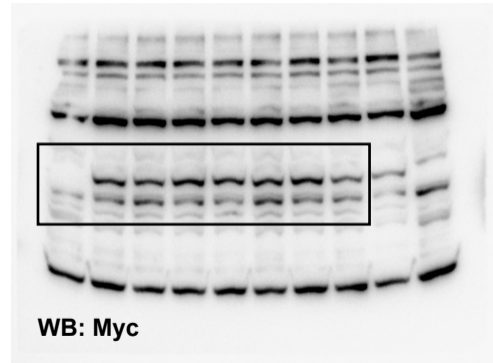

Supplement: Figure 7—figure supplement 1—source data 1. — Relevant bands are inside rectangles. EGFP bands also visible on Myc immunoblot on the left. See Figure 7—figure supplement 1 for more details. [file elife-78383-fig7-figsupp1-data1.pdf]

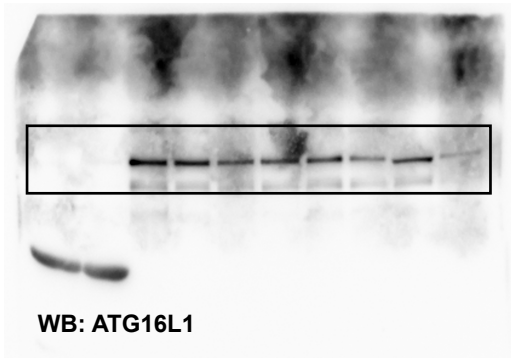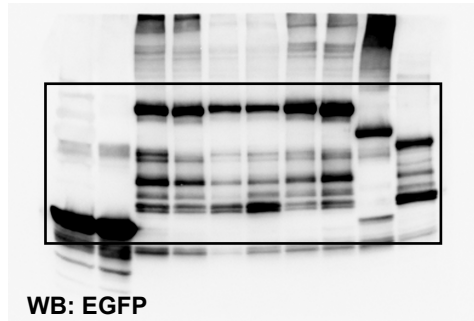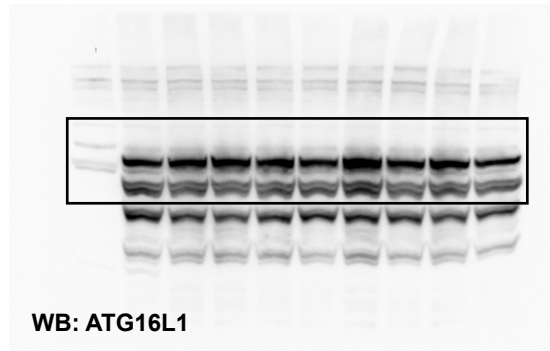

Supplement: Figure 8—source data 1. — Relevant bands are inside rectangles. See Figure 8 for more details. [file elife-78383-fig8-data1.pdf]
